# Self-rated health and its determinants in patients with hypertension in Isfahan in 2019

**DOI:** 10.1186/s12889-024-17887-2

**Published:** 2024-02-15

**Authors:** Asieh Mansouri, Alireza Khosravi Farsani, Noushin Mohammadifard, Fatemeh Nouri, Mahnaz Jozan, Ghazaal Alavi Tabatabaei, Rezvan Salehidoost, Hamed Rafiee

**Affiliations:** 1 Hypertension Research Center, Cardiovascular Research Institute, Isfahan University of Medical Sciences, Isfahan, Iran; 2 Department of Biostatistics and Epidemiology, School of Health, Isfahan University of Medical Sciences, Isfahan, Iran; 3 Isfahan Cardiovascular Research Center, Cardiovascular Research Institute, Isfahan University of Medical Sciences, Isfahan, Iran; 4 Centre for Endocrinology, William Harvey Research Institute, Barts and The London School of Medicine and Dentistry, Queen Mary University of London, London, UK; 5 Cardiac Rehabilitation Research Center, Cardiovascular Research Institute, Isfahan University of Medical Sciences, Isfahan, Iran

**Keywords:** Self-rated health, Hypertension, Socioeconomic status, Chronic disease, Iran

## Abstract

**Background and objectives:**

Self-rated health (SRH) serves as an assessment of contentment regarding one’s social, mental, and physical well-being and has been linked to both cardiovascular mortality and morbidity. Nonetheless, the relationship between SRH and medical outcomes in individuals with hypertension unsettled. This research endeavors to pinpoint the determinants that affect SRH in Iranian patients with hypertension.

**Materials and methods:**

This cross-sectional study took place in Isfahan, Iran, from November 2018 to August 2019 and involved 886 patients with essential HTN. The data collection methods included a checklist for demographic information and risk factors, blood pressure measurements (systolic and diastolic), the Persian version of the 8-Item Morisky Medication Adherence scale, and a self-rated health questionnaire recommended by the World Health Organization. Independent sample T-test and chi squared test were used for comparison of variables between two groups of SRH. Additionally, multivariable logistic regression was used to analyze the factors influencing self-rated health status.

**Results:**

Among 886 participants (mean age 57.8 ± 8.8 years, 71.9% women), 89.62% reported good SRH. Comorbid conditions were significantly associated with poorer SRH (*p* < 0.05). Notably, higher education (odd ratio (OR) = 1.88, 95% confidence interval (CI) = 1.13–3.11, *p* = 0.015) and increased income (OR = 4.34, 95% CI = 1.43–13.18, *p* = 0.010) were identified as positive determinants of good SRH.

**Conclusion:**

We concluded that socioeconomic factors (education and income) and comorbid conditions (diabetes, hyperlipidemia, and pulmonary diseases) are risk factors for poor SRH among hypertensive patients. These findings could help planning of health enhancement initiative.

## Introduction

A person’s level of satisfaction with various aspects of their social, mental, and physical health is known as their “self-rated health” (SRH) [1]. As a result of SRH’s simplicity, reliability, validity, and putative biological basis, it has been frequently employed in public health research [2–4]. Large-scale studies have highlighted that SRH is closely correlated to cardiovascular health outcomes [5], morbidity, and mortality in people [4]. SRH’s results indicate a wide range of variability across different ethnicities, countries, and also genders, which is partly due to the impact of demographic variables (e.g., age and sex), lifestyle, physical activity, income, and education of individuals [6]. Additionally, another important factor affecting SRH and patients’ quality of life is the presence of chronic disease [7]. One of the most significant chronic diseases is hypertension (HTN), which not only has significance as a standalone condition but also considerably influences the likelihood of developing other noncommunicable diseases, such as cardiovascular disease, cerebrovascular disease, and chronic kidney disease [8]. From 1990 to 2019, there has been a twofold increase in global HTN cases [9]. Present statistics from Iran indicate rates of around 25% in the general population and 42% among older individuals which despite advancements in treatment and technology, no decrease in HTN rates is seen between 2014 and 2017 [10]..This disease is considered the first priority in cardiovascular research in Iran [11]. Moreover, HTN enormously has influenced the economy around the world, especially in Middle East countries enormously [12, 13]. The estimated prevalence of HTN in the Middle East and North Africa is 26.2% according to a recent meta-analysis of 147 studies with an increasing trend over the past decades [14]. However, awareness and treatment have not improved notably [12, 15]. Therefore, early interventions are essential to reduce the deleterious effects of HTN and its accompanying complications.

Several recent endeavors have evaluated the relationship between blood pressure (BP), SRH, and overall quality of life. For instance, in a study conducted on Chinese people, there was a substantial link between controlled BP and excellent SRH [8]. Furthermore, poor control of HTN and suboptimal SRH score in Australian patients were associated with a higher frequency of visiting general practitioners monthly [16]. Although SRH’s determinants have been evaluated in some nations; studies are scarce regarding SRH and its determinants in hypertensive patients in Iran. As a result, we aimed to determine SRH and its determinants in Iranian patients with diagnosed HTN. This research has the potential to pave the way for significant improvements in HTN treatment and prevention in Middle Eastern countries.

## Methods

### Study design and population

This study was a cross-sectional study conducted between November 2018 to August 2019 in Isfahan province, Iran. The study population consisted of 886 men and women with essential HTN who were registered in Isfahan’s Comprehensive Health Service Centers (CHSCs). These patients were selected using a multistage sampling procedure. Considering geographical distribution and the total number of patients with HTN registered in each center we selected 15 centers among whole Isfahan CHSCs. After preparing the list of all patients at each center and regarding calculated total sample size, we determined the number of patients needed from each center proportionally to its total number of registered patients with HTN. Finally, we applied simple random sampling (using the Microsoft Excel program) to select subjects from the entire list of patients in that center. Inclusion criteria included an age between 30 and 70 years old, a diagnosis of essential HTN, and taking at least one anti-hypertensive medication. Our exclusion criteria included being over 70 or under 30, suffering from an incurable illness (such as cancer in an advanced stage), having mental retardation, or lack of agreement to participate in the study. Informed consent was obtained from all patients participating in the study. The current study was approved by the Ethics Committee of Isfahan University of Medical Sciences.

### Sample size

The formula ($$ n= \frac{{({Z}_{1-a/2}+{Z}_{1-B})}^{2} {S}_{d}^{2}}{{\stackrel{-}{d}}^{2}}$$) was used for sample size calculation. Considering α = 0.05 and β = 0.2, $$ {S}_{d}=0.104$$ and $$ \stackrel{-}{d}=0.011$$ according to Jaffe et al. [17] and a loss to follow-up of 20%, total sample size was calculated as 876.

### Data collection tools

The data collection tools used in primary study included (1) a checklist of demographic information (e.g. age, gender, education, employment, monthly income and marital status); risk factors information (e.g. dyslipidemia, diabetes, pulmonary disease, using antihypertensive drugs regularly and HTN control); BP values (the mean systolic and diastolic BP (mmHg)), (2) Persian version of the 8-Item Morisky Medication Adherence scale validated by Moharramzad et al. [18]. and (3) SRH that was asked by a 5-point scaled question recommended by the World Health Organization [19] “In general, how would you describe your current state of health: bad, very bad, intermediate, good, very good ”. We dichotomized SRH into 2 categories good (comprising of the answers intermediate, good, and very good) and poor (comprising the answers bad and very bad) in line with other studies [20, 21].

### Statistical analysis

We used IBM SPSS 25 software for data analysis. Quantitative and qualitative variables were described using mean (standard deviation (SD)) and number (%), respectively. A comparison of quantitative and qualitative variables between patients with good or poor SRH was performed using the independent sample T-test and chi-square test, respectively. To identify predictors of SRH in patients with HTN we used logistic regression. We applied the forward strategy, recommended by Hosmer and Lemeshow [22] to determine SRH predictors. In the first step, we ran a univariate logistic regression for age, sex, education, employment, marital status, income, diabetes, dyslipidemia, pulmonary disease, HTN control, taking antihypertensive medicines and compliance with treatment, one by one. Then, we entered all variables with a P value below 0.2 in the first step in a multivariable logistic regression. Variables with a P value below 0.05 remained in the final model.

## Results

### Sample characteristics

Eight hundred eighty-six subjects attended the study, of which 71.9% were female. The mean age of the participants was 57.8 ± 8.8 (range 32–84). Among the whole sample, 571 (87.4%) were married, and 82 (12.6%) were unmarried. Most of the patients had nonacademic education (71.9%) and a monthly income below < 20,000,000 IRR (84.7%). Three hundred eighty-one (43.7%) patients had diabetes. Further details are presented in Table 1.

**Table 1:** characteristics of Participants in total and by self-rated health status

Variable		n (%) / mean (SD)
Age		57.8 (8.8)
Gender (male)		249 (28.2)
Education	Illiterate	164 (18.6)
	Non-academic education	636 (71.9)
	Academic education	84 (9.5)
Employment	Employed	122 (13.8)
	Unemployed	762 (86.2)
Marital status	Married	571 (87.4)
	Unmarried	82 (12.6)
Monthly income	< 20,000,000 IRR	749 (84.7)
	≥ 20,000,000 IRR	135 (15.3)
Diabetes (yes)		381 (43.7)
Dyslipidemia (yes)		516 (59.9)
Pulmonary disease (yes)		70 (8.0)
Systolic blood pressure		133.2 (16.6)
Diastolic blood pressure		78.7 (10.5)
HTN control (yes)		338 (38.5)
Taking anti-hypertensive medicine	Regular	831 (94.1)
	Irregular	48 (5.4)
	No treatment	4 (0.5)
Compliance to treatment	Low	224 (25.5)
	Intermediate	315 (35.9)
	High	339 (38.6)

IRR: Iranian Rial

*P* < 0.05 was considered significant

Values are presented as mean (SD) for continuous variables and n (%) for categorical variables

### Description of SRH by exploratory variables

Seven hundred ninety-four patients (89.62%) rated their health as good, and 90 (10.15%) patients reported poor SRH. Two subjects did not answer this question. The comparison of participants with good and poor SRH in terms of demographic and clinical characteristics is displayed in Table 2. We didn’t observe any significant relationship between SRH rating and age and sex. However, the SRH distribution was significantly different among different levels of education (*p* = 0.005), as the illiterate participants rated their health lower than the educated group (17.1% poor SRH vs. 8.6% and 8.3%). On the contrary to employment status, monthly income had a significant association with SRH (*p* = 0.003); High-income patients reported higher levels of SRH (97.0% good SRH in the high-income group vs. 88.5% in the low-income group). A good SRH was more prevalent in older, male, married, and employed patients; however, these differences were not statistically significant. Patients with a history of diabetes, dyslipidemia, and pulmonary disease, significantly rated their SRH lower than those without these diseases. (*p* < 0.001, *P* = 0.008, and *P* = 0.041, respectively). Whether the patient’s BP was controlled, or how much the patient was compliant to the hypertension treatment did not display any significant association with the SRH rating.

**Table 2:** Description of self-rated health by exploratory variables

Variable		SRH (%)		p-value
		Good SRH	Poor SRH	
Age	≤ 60 years	465 (90.6)	48 (9.4)	0.341†
	> 60 years	329 (88.7)	42 (11.3)	
Gender	Male	230 (92.4)	19 (7.6)	0.116†
	Female	564 (88.8)	71 (11.2)	
Education	Illiterate	136 (82.9)	28 (17.1)	0.005†
	Non-academic education	581 (91.4)	55 (8.6)	
	Academic education	77 (91.7)	7 (8.3)	
Employment	Employed	111 (91.0)	11 (9.0)	0.647†
	Unemployed	683 (89.6)	79 (10.4)	
Marital status	Married	512 (89.7)	59 (10.3)	0.136†
	Unmarried	69 (84.1)	13 (15.9)	
Monthly income	< 20,000,000 IRR	663 (88.5)	86 (11.5)	0.003†
	≥ 20,000,000 IRR	131 (97.0)	4 (3.0)	
Diabetes	Yes	327 (85.8)	54 (14.2)	< 0.001†
	No	456 (93.1)	34 (6.9)	
Dyslipidemia	Yes	453 (87.8)	63 (12.2)	0.008†
	No	322 (93.3)	23 (6.7)	
Pulmonary disease	Yes	58 (82.9)	12 (17.1)	0.041†
	No	727 (90.5)	76 (9.5)	
hypertension control	Yes	490 (90.7)	50 (9.3)	0.221†
	No	298 (88.2)	40 (11.8)	
Taking anti-hypertensive medicine	Regular	746 (89.8)	85 (10.2)	0.597‡
	Irregular	44 (91.7)	4 (8.3)	
	No treatment	3 (75.0)	1 (25.0)	
Compliance to treatment	Low	199 (88.8)	25 (11.2)	0.551†
	Intermediate	288 (91.4)	27 (8.6)	
	High	303 (89.4)	36 (10.6)	

IRR: Iranian Rial

† Chi-square Test

‡ Fisher Exact Test

*P* < 0.05 was considered significant

### Determinants of SRH

We used forward logistic regression to identify SRH determinants in patients with hypertension. As revealed in Table 3, among demographic variables, education level and income had significant associations with SRH, i.e., educated patients without university degrees (odd ratio (OR) = 1.88, 95% confidence interval (CI) = 1.13–3.11, *p* = 0.015) and patients earning more than twenty million Rials per month (OR = 4.34, 95% CI = 1.43–13.18, *p* = 0.010) were more likely to report good SRH. Among the comorbidity variables, not having diabetes was associated with higher SRH (OR = 2.12, 95% CI = 1.34–3.35, *p* = 0.001).

**Table 3:** Predictors of good SRH

variable		95% confidence interval	P-value
comorbidities			
Having diabetes	1	-	-
No diabetes	2.12	1.34, 3.35	0.001
education level			
Illiterate	1	-	-
Non-academic education	1.88	1.13, 3.11	0.015
Academic education	1.01	0.38, 2.68	0.98
monthly income			
≤ 20,000,000 IRR	1	-	-
> 20,000,000 IRR	4.34	1.43, 13.18	0.01

*P* < 0.5 was considered significant

Model details:

Pseudo R2 = 0.0509, Chi-square of likelihood ratio test = 29.02; *P* < 0.001

## Discussion

In the present study of 886 hypertensive patients, we determined the status of SRH in patients with hypertension and investigated the association of demographic variables and comorbidities with good and poor SRH. In this study nearly 90% of patients rated their health status as good. In terms of demographic variables, higher income, and education level were associated with a good rating of SRH, whereas such associations were not observed for age, sex, marital status, and employment status. Among comorbidities, diabetes, dyslipidemia, and pulmonary diseases were associated with poor SRH. We also observed that preuniversity education and high income increased the odds of good SRH by roughly two and four times, respectively.

The distribution of SRH ratings has exhibited considerable variability in prior literature across diverse populations. For instance, a cross-sectional investigation involving 4,860 hypertensive individuals aged 65 years and older in China revealed that approximately 60% of the participants assessed their health as suboptimal [23]. In another study conducted in China, roughly 40% of 807 individuals with hypertension reported poor health status [8]. Conversely, in a study encompassing 942 elderly patients aged 60 years or older with diabetes and/or hypertension, an impressive 80% of participants reported good health [24]. In our present study, an overwhelming majority, approximately 90% of the study sample, provided favorable assessments of their health status.

The variation in SRH distribution can be attributed to the multifaceted nature of SRH, which is influenced by a combination of psychological, social, and physical factors. These factors exhibit significant diversity among different populations, thus providing a justifiable explanation for the observed inconsistency in SRH ratings.

In the present study, we did not observe any significant differences in SRH between men and women. The relationship between gender and SRH has demonstrated a high degree of variability in previous literature. While some investigations reported higher SRH ratings among men [25, 26] others have found the opposite trend [26, 27], and certain studies have even indicated no discernible gender-associated disparity [28].

The absence of a substantial gender-based discrepancy in SRH within our study could be attributed to several factors. Firstly, it is plausible that gender-related health disparities have evolved or diminished in recent years, reflecting shifts in societal attitudes and improved healthcare access. These changes may have contributed to a more equitable perception of health between genders, reducing the magnitude of gender-based differences. Secondly, it is important to acknowledge that our study may have been underpowered to detect subtle gender differences in SRH, should they exist. The sample size, while sufficient for our primary analyses, might not have been optimal for exploring nuanced variations between men and women. Consequently, future research endeavors should consider larger and more diverse samples to enhance statistical power. Finally, it is conceivable that other determinants, such as socioeconomic status, education, or cultural factors, exerted a more pronounced influence on SRH within our sample than gender.

In our analysis, we did not find any significant relationship between marital status and SRH. This finding contrasts with certain prior investigations. The disparity in our observations can be attributed to two overarching theoretical frameworks: the “marriage selection” and “marriage protection” paradigms [29].

The “marriage selection” theory posits that individuals in better health are more likely to enter into marriage, thereby generating the apparent association between marriage and improved SRH. Conversely, the “marriage protection” theory posits that marriage exerts a positive influence on various factors, including social support, economic well-being, risk behavior prevention, and overall mortality and morbidity [29], which results in better SRH among married individuals.

Our findings may be indicative of the susceptibility of these two mechanisms to cultural variations. In the context of Iranian culture, it is plausible that there are no significant disparities in the health status of individuals who choose to marry and those who do not, and a similar pattern might hold for the “marriage protection” theory.

The observed association between SRH and socioeconomic factors, specifically income and education levels, aligns with findings from prior research [30–32]. The interplay between education levels and good SRH may be attributed to bidirectional causality, where higher education positively influences health, and improved health, in turn, facilitates educational attainment. This duality has been explored in the literature, with some studies providing support for the latter hypothesis. These investigations suggest that individuals in better health are more likely to achieve higher levels of education, with potential confounding factors, such as the quality of parental care [33], impacting both health and educational outcomes. Nonetheless, a more substantial body of evidence lends credence to the causal effect of education on health [34] This theory posits that higher education exerts a favorable influence on various determinants of health, including income, lifestyle choices, access to social resources and healthcare, cognitive abilities, and skills pertinent to health management [34].

In alignment with previous research [35–37], our study demonstrates a consistent association between income and SRH. Prior investigations have consistently reported that individuals with lower income levels tend to face barriers to accessing healthcare services, engage in less physical activity, pay limited attention to other aspects of a health-related lifestyle, and consequently experience poorer health outcomes compared to their higher-income counterparts [38–41] Furthermore, some studies have posited that the adverse self-rated health reported by individuals with lower incomes may, in part, be influenced by their perception of socio-economic deprivation, which can skew their evaluation of their overall health status [42–44].

In line with prior research [45], our study supports the association between chronic comorbid conditions and low SRH. The impact of chronic diseases on SRH may be influenced by various interconnected factors, including the potential mediating role of psychological variables such as self-esteem, self-worth, and self-mastery, which relates to an individual’s perceived control over their life circumstances. Notably, self-mastery, a valuable resource for coping with stressful situations [46], emerged as a significant predictor of diminished SRH among individuals dealing with chronic conditions, as observed in a study by Cott et al. [47]

The observed link between diabetes and SRH in our study is congruent with findings in the existing literature [45]. This association has been substantiated by the adverse impact of diabetes complications on daily activities [48] The impairment of daily activities was noted to be more pronounced in patients of advanced age, with longer diabetes duration, elevated fasting glucose levels, severe obesity, insulin dependency, and concurrent hypertension [49]. Furthermore, an investigation involving 1837 adults with type 2 diabetes revealed that disability and depression were predictive factors for diminished SRH [50]. These findings collectively underscore the multifaceted nature of the relationship between diabetes and SRH [25–28].

Similar to the observed association with diabetes, our study aligns with previous research in confirming a relationship between pulmonary diseases and diminished SRH. A study involving 8200 adults found that, even after accounting for potential confounding variables, having asthma was significantly associated with poor SRH. Several factors may help explain this association, including suboptimal asthma control, anxiety related to disease exacerbation, and the influence of inflammatory cytokines [51].

Moreover, the association between poor SRH and various pulmonary diseases, such as chronic obstructive pulmonary disease (COPD) and interstitial lung disease, has been consistently reported [52, 53]. Moreover, the association between poor SRH and various pulmonary diseases, such as COPD and interstitial lung disease, has been consistently reported [54].

The primary limitation of our study arises from its cross-sectional design, which precludes the establishment of causal relationships. Additionally, a noteworthy limitation pertains to the predictive power of the determinants included in our logistic model with respect to SRH. This limitation may be attributed to the nature of the data we utilized, which were not primarily collected for the explicit purpose of assessing SRH. Consequently, we recommend the implementation of a large-scale, preferably longitudinal, investigation specifically aimed at rigorously measuring SRH and its determinants in patients with hypertension.

It is worth highlighting that our study’s sampling methodology represents a notable strength, as it was thoughtfully designed to secure a sample that adequately reflects the broader Iranian population.

### Conclusion and recommendations

In the present study, significant associations were observed between SRH and socioeconomic variables such as education level and income in hypertensive individuals, alongside a link to comorbid conditions like diabetes, hyperlipidemia, and pulmonary diseases. In contrast, SRH did not significantly correlate with age, sex, or marital status.

Given the constraints of the cross-sectional design, prospective longitudinal studies are warranted to confirm causality and further elucidate the psychosocial factors affecting SRH disparities. Although no definitive association was found between hypertension management and SRH, the data indicated atypical SRH trends in patients with variable medication adherence, prompting calls for further research with larger cohorts.

Implications for healthcare policy from these findings suggest prioritizing educational and economic enhancements and addressing comorbidities to improve SRH. The absence of gender differences in SRH could guide the development of more unified and potentially more cost-effective health promotion strategies for both genders.

## Data Availability

The data that support the findings of this study are available from [Asieh Mansouri] but restrictions apply to the availability of these data, which were used under license for the current study, and so are not publicly available. Data are however available from the authors upon reasonable request and with permission of [Asieh Mansouri].

## Abbreviations

SRH: Self-rated health
HTN: Hypertension
BP: Blood pressure
SD: Standard deviation
COPD: Chronic obstructive pulmonary disease
CI: Confidence interval
OR: Odd ratio
